# Meiotic Silencing in Dothideomycetous *Bipolaris maydis*

**DOI:** 10.3389/ffunb.2022.931888

**Published:** 2022-06-27

**Authors:** Kenya Tsuji, Yuki Kitade, Akira Yoshimi, Chihiro Tanaka

**Affiliations:** ^1^Laboratory of Terrestrial Microbiology, Graduate School of Agriculture, Kyoto University, Kyoto, Japan; ^2^Laboratory of Environmental Interface Technology of Filamentous Fungi, Graduate School of Agriculture, Kyoto University, Kyoto, Japan; ^3^Terrestrial Microbiology and Systematics, Global Environmental Studies, Kyoto University, Kyoto, Japan

**Keywords:** meiotic silencing, meiotic silencing by unpaired DNA, *Bipolaris maydis*, *Cochliobolus heterostrophus*, septin, ascospore formation

## Abstract

The filamentous ascomycete *Bipolaris maydis* is a plant pathogen that causes corn leaf blight and has been used in cytological studies of sexual reproduction. In this fungus, when null mutants of each septin are crossed with the wild-type strain, all ascospores derived from the same asci show abnormal morphology. The phenomenon was remarkably similar to the event known as “ascus dominance” in *Neurospora crassa*, which is known to be caused by MSUD (meiotic silencing by unpaired DNA). However, it is not clear whether *B. maydis* possesses functional MSUD. The object of this study is to elucidate whether this fungus carries a functional MSUD system that causes ascus dominance in the crosses of septin mutants and the wild-type strain. The results of homozygous and heterozygous crossing tests with mutants, having the insertional *CDC10*-septin gene sequence into the genome, suggested that the ascus dominance in *B. maydis* is triggered by the unpaired DNA as in *N. crassa*. To investigate whether MSUD is caused by the same mechanism as in *N. crassa*, an RNA-dependent RNA polymerase, one of the essential factors in MSUD, was identified and disrupted (Δ*rdr1*) in *B. maydis*. When the Δ*rdr1* strain was crossed with each mutant of the septins, ascus dominance did not occur in all crosses. These results suggest that this ascus dominance is caused by RNA silencing triggered by an unpaired gene, as in *N. crassa*, and septin genes were affected by this silencing. To date, although MSUD has been found only in *Fusarium graminearum* and *N. crassa*, which are classified as Sordariomycetes, this study showed that MSUD is also functional in *B. maydis*, which is classified as a Dothideomycete. These results showed the possibility that this posttranscriptional regulation is extensively conserved among filamentous ascomycetes.

## Introduction

Ascospores are the offspring formed through the process of sexual reproduction in ascomycetes and are developed in the ascus, a specialized cell for sexual reproduction. Ascospores in a single ascus inherit each parental allele and the segregation ratio of parental alleles shows 1:1. Morphological phenotype of the ascospores is generally determined by the alleles they possess (Raju, 1994). However, sometimes there are the phenomena that the morphology of ascospores does not depend on the alleles possessed (Davidow et al., 1980; Aramayo et al., 1996). One of those phenomena is the “Ascus dominance” (Aramayo et al., 1996).

Ascus dominance has been studied extensively in disruption mutants of the *Asm-1* gene, which is involved in maturation of the ascospore in *Neurospora crassa* (Aramayo et al., 1996). When this null mutant of *Asm-1* is crossed with the wild-type strain, almost all the ascospores in a single ascus are immature and not the normal black color. The phenotype of the wild-type allele is suppressed and the phenotype of the null allele predominantly appears in the ascus. Shiu et al. (2001) studied this phenomenon in which all ascospores fail to mature and discovered that it is caused by meiotic silencing by unpaired DNA (MSUD; Shiu et al., 2001). MSUD is posttranscriptional regulation that causes the silencing of genes that are not paired properly in the genome during nuclear fusion. When the null strain of *Asm-1* is crossed with the wild-type strain, *Asm-1* is silenced during meiosis due to unpaired null and wild-type alleles. When the *Asm-1* gene is inserted ectopically in the genome, the normally paired *Asm-1* gene is also silenced (Aramayo and Metzenberg, 1996). This silencing occurs specifically during karyogamy and ascospore formation and if the silenced gene is required for meiosis and ascospore formation, an abnormal phenotype will appear in all ascospores within the ascus, resulting in ascus dominance. The function of MSUD is based on the mechanism of RNA interference (RNAi) and the RNA-dependent RNA polymerase (RdRP) is indispensable to MSUD (Shiu et al., 2001; Son et al., 2011). Lack of the RdRP gene contributes to the suppression of MSUD (Shiu et al., 2001).

**Figure 1:**
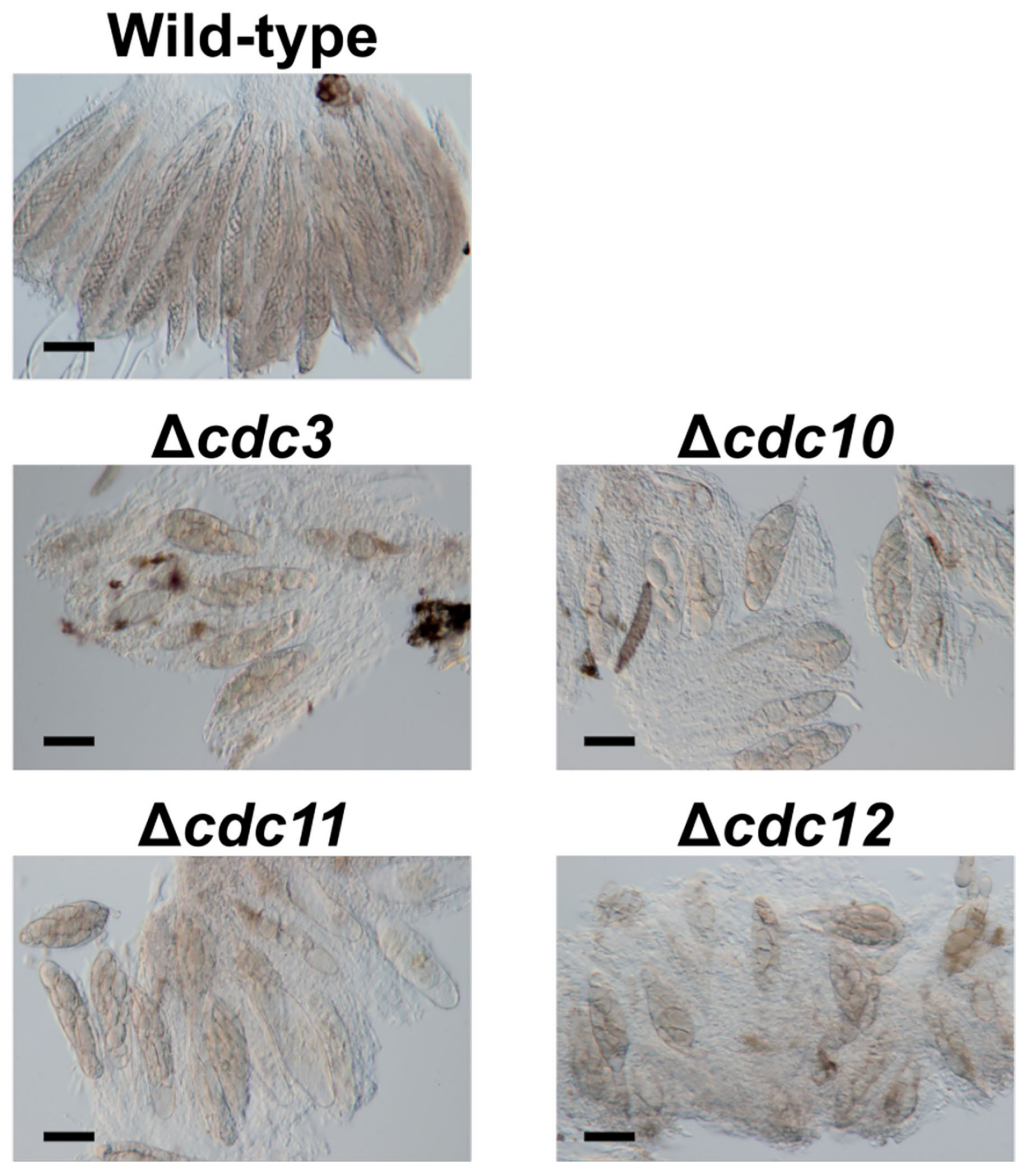
Asci and ascospores from crosses between the null mutants of core septin genes and the wild-type strain. The null mutants of core septin genes were crossed with the wild-type strain. After 4 weeks, formed pseudothecia were harvested and dissected to observe asci and ascospores. *Bars*: 50 µm.

*Bipolaris maydis* (syn. *Cochliobolus heterostrophus*, belonging to the class of Dothideomycetes) is a necrotrophic pathogen that causes southern corn leaf blight. This fungus has a heterothallic mating system and easily mates between strains with the opposite mating type (*MAT1-1* and *MAT1-2*) under laboratory conditions. Thus, this fungus is also used in cytological studies of sexual reproduction, as in the case of *N. crassa* (Turgeon, 1998; Raju, 2008; Sumita et al., 2017). In a previous study, *CLA4* encoding a PAK-like kinase was characterized and it was clarified that *CLA4* is involved in ascospore development (Kitade et al., 2019). To study the molecular base of sexual reproduction in this fungus, we have generated disruption mutants of four core septin genes (orthologues to *Saccharomyces cerevisiae CDC3*, *CDC10*, *CDC11*, and *CDC12*), whose products might interact with Cla4. When these disruption strains were crossed with the wild-type strain, all ascospores derived from a single ascus showed abnormal morphology (unpublished data, q.v., **Figure 1**). This phenomenon was remarkably similar to the ascus dominance in *N. crassa*. As far as we know, MSUD, the main cause of ascus dominance, has been found in only two fungi, *N. crassa* and *Fusarium graminearum* (Shiu et al., 2001; Son et al., 2011). While these two fungi are categorized as Sordariomycetes, *B. maydis* is categorized as a Dothideomycete. To date, it is not clear whether a fungus not belonging to the Sordariomycete class possesses functional MSUD.

In this study, we investigated whether MSUD is responsible for the abnormal morphology of ascospores derived from the cross between the core septin mutants and the wild-type strain. We constructed several mutants by ectopic or entopic insertion of the *CDC10*-gene sequence and inspected ascospores from the heterozygous cross between these mutants and the wild-type strain. We identified the *B. maydis RDR1* gene which encodes putative MSUD-related RdRP by phylogenetic analysis and generated an RDR1 disruption (Δ*rdr1*) strain derived from the wild-type strain. We crossed the Δ*rdr1* strain and each core septin mutant and observed ascospore from these crosses.

## Materials and Methods

### Strains and Culture Conditions

*Bipolaris maydis* HITO7711 (*MAT1-2*) and MASIKI2-2 (*MAT1-1*) were used as wild-type strains throughout this study. Fungal mutants in this study were generated from HITO7711 and MASHIKI2-2. All strains were maintained on a complete medium agar (CMA; Tanaka et al., 1991) or V8 agar (V8A; Ribeiro, 1978) at 25°C. A clarified V8 juice agar (CV8A; Erwin and McCormick, 1971) was used to measure colonial growth.

### Generation of Septin Gene Disruption Mutants

All primers used in this study are listed in **Supplementary Table S1**. Molecular experiments were performed according to Sambrook et al. (1989). For the generation of null mutants, we adopted a targeted replacement method using homologous recombination. The genomic DNA of *B. maydis* HITO7711 was obtained *via* the protocol described by Izumitsu et al. (2012). The disruption cassette of the *CDC10* gene was constructed *via* PCR fusion (Szewczyk et al., 2006). We replaced the *CDC10* gene with a hygromycin B phosphotransferase (*HPH*) cassette obtained from the plasmid pCB1004 (Carroll et al., 1994). In the first-round PCR, the 5′- and 3′-flanking regions of the *CDC10* gene were amplified from genomic DNA of the wild-type HITO7711 using ExTaq polymerase (Takara Bio, Kusatsu, Japan) and primer sets (Cdc10-f1/Cdc10-r1 for the 5′-flanking region and Cdc10-f2/Cdc10-r2 for the 3′-flanking region). The *HPH* cassette was also amplified from the plasmid pCB1004 using ExTaq polymerase and primers pCB1004-f1 and pCB1004-r1. These three DNA fragments were used as substrates for the second-round PCR in order to fuse the three fragments into one deletion cassette using primers Cdc10-f1 and Cdc10-r2. The resulting major PCR product was purified through ethanol precipitation and transformed into protoplasts of the wild-type strains, HITO7711 and MASHIKI2-2. Transformation experiments were performed using the method described by Izumitsu et al. (2009). Integration of the deletion cassette was confirmed using the PCR method with three primer sets (Cdc10-f3/HPH-chk-f1, Cdc10-f4/Cdc10-r4, and HPH-chk-r1/Cdc10-r3). The same procedure, except for the primer sets, was adopted to generate disruption mutants of the *CDC3*, *CDC11*, and *CDC12* genes.

### Generation of the Complement Strain and *CDC10* Frameshift Mutant

For functional complementation of the *CDC10* disruption mutant, we constructed a plasmid vector with the complete *CDC10* gene. We amplified a fragment of the *CDC10* gene containing 5′- and 3′-flanking regions from the genomic DNA of the wild-type HITO7711 with primers Cdc10-PG-f1 and Cdc10-PG-r1 using PrimeSTAR GXL DNA-polymerase (Takara Bio). We used a NEBuilder HiFi DNA Assembly Master Mix (New England Biolabs, Ipswich, USA) and DNA Ligation Kit Ver. 2.1 (Takara Bio) to construct the plasmid. The amplified fragment was ligated into the *Sca*I site of the plasmid pZGenI (Sumita et al., 2017) carrying a neomycin phosphotransferase II gene (*NPTII*) using the HiFi DNA Assembly method. Next, the 3′-flanking region of the *CDC10* gene was amplified using the primer set Cdc10r-f1/Cdc10r-r1. The amplified fragment was ligated into the *Pme*I site of the constructed plasmid. The resulting plasmid was designated as pZGCDC10C.

To ensure pairing of *CDC10* alleles during meiosis, we generated a mutant with a frameshift mutation in *CDC10* ORF. To construct a *CDC10* fragment harboring the mutation, two fragments of the *CDC10* gene containing the 5′- and 3′-flanking regions were amplified from the genomic DNA of the wild-type HITO7711 with primer sets Cdc10-PG-f1/Cdc10-PGm-r1 and Cdc10-PGm-f1/Cdc10-PG-r1, respectively. The Cdc10-PGm-f1 primer was used to introduce a single nucleotide deletion. The resulting fragments were inserted into the *Sca*I site of pZGenI using the HiFi DNA Assembly method. Next, the 3′-flanking region of the *CDC10* gene was amplified using the primer set Cdc10r-f1/Cdc10r-r1. The amplified fragment was ligated into the *Pme*I site of the constructed plasmid. The resulting plasmid was verified for the deletion of a single base pair in the *CDC10* ORF, *via* sequencing analysis, and designated as pZGCDC10fs. In this *CDC10* gene, cytosine at the 31^st^ nucleotide from the start codon was deleted so that a truncated peptide with 48 amino acid residues (regular 10 residues + irregular 38 residues) would be translated in a fungal transformant.

Two plasmids, pZGCDC10c and pZGCDC10fs, were digested with the *Xba*I for fungal transformation. Transformations of these two cassettes in the Δ*cdc10* protoplasts were performed through a double crossover event of homologous recombination within the 5′- and 3′-flanking regions of the *CDC10* gene. Transformation experiments were performed using the method described by Izumitsu et al. (2009). The transformants were selected with 300 ppm (w/v) geneticin. In addition, the correct plasmid integrations were confirmed by PCR with primer sets Cdc10-f5/Cdc10-r5, Cdc10-f4/Cdc10-r4, and Cdc10-f6/Cdc10-r6. The resulting strains were designated as a reconstituted strain (*CDC10^comp^*) and a frameshift mutant strain (*cdc10^fs^*). In addition, we obtained another reconstituted strain (*CDC10^ect^*) in which the reconstitution cassette was integrated ectopically into the Δ*cdc10* protoplasts. In a similar procedure, we also generated transformants (*CDC10^fsect^*) with a frameshift cassette, introduced ectopically, into the wild-type protoplasts.

### Morphology of Colonies and Conidia

To investigate radial growth, each strain was inoculated on CV8A in 90 mm petri dishes and cultured at 25°C in darkness. After 7 days, the colony diameter was measured. The colony diameter of each strain was presented as the average of two locations and the measurements were repeated on three individual plates. To investigate the morphology of conidia, conidia of each strain were collected from a 7 day-old colony on V8A. They were fixed with 5% (v/v) glutaraldehyde at 4°C for 1 day and washed with a phosphate buffer. These samples were stained with 20 µg/ml Calcofluor White (CFW; Sigma-Aldrich, Saint Louis, USA) to visualize septa. Conidia, stained with CFW, was observed using DIC-epifluorescent microscopy (Leica DML with A cube (BP 340–380 195 nm excitation filter, 400 nm dichromatic mirror, LP 425 nm suppression filter; Leica Microsystems, Wetzler, Germany).

### Microscopy for Ascospores

Crossing was performed on a sterilized corn leaf on Sachs’s agar medium according to the method described by Tanaka et al. (1991). The compatible strains with different mating types were inoculated at opposite sides of a maize leaf. These cultures were incubated at 25°C for 4 weeks in darkness. In order to observe the ascospores, mature pseudothecia were harvested and dissected in 10% glycerol on glass slides. Subsequently, these asci and ascospores were observed under a microscope Leica DML.

### Identification of RNA-Dependent RNA Polymerase for MSUD in *B. maydis*

To find the RdRP genes in *B. maydis*, BLASTp analysis was performed against the reference genome of the *B. maydis* C5 strain at the NCBI website. The amino acid sequence of the conserved region (RdRP domain: 707–1083 aa) in *N. crassa* Qde1 (NCU07534) was used as a query. The word size was changed from 6 to 3 and the other parameters were left unchanged.

To determine the RdRP gene for MSUD in *B. maydis*, phylogenetic analysis was performed. The deduced protein sequences of the RdRP in filamentous fungi were obtained from the NCBI website. The locus tag of each candidate protein in this analysis is listed in **Supplementary Table S2**. The RdRP domain in the *N. crassa* Qde1 protein was used for the analysis to obtain multiple alignment and a phylogenetic tree. The domain was retrieved from the Pfam database (Finn et al., 2016). The amino acid sequence in several filamentous fungi was aligned with MUSCLE (Edgar, 2004). A phylogenetic tree was constructed using the NJ (neighbor-joining) method with the default parameter using MEGA X (Kumar et al., 2018).

### Generation of *RDR1* Disruption Mutants

Gene disruption was performed using the same method as above. We replaced the *RDR1* gene with an *NPTII* cassette obtained from the plasmid pZGenI. In the first-round PCR, the 5′- and 3′-flanking regions of the *RDR1* gene were amplified from genomic DNA of the wild-type HITO7711 using ExTaq polymerase and primer sets (Rdr1-f1/Rdr1-r1 for the 5′-flanking region; Rdr1-f2/Rdr1-r2 for the 3′-flanking region). The *NPTII* cassette was also amplified from the plasmid pZGenI using ExTaq polymerase and primers Gen-f1 and Gen-r1. These three DNA fragments were used as substrates for the second round of PCR in order to fuse the three fragments into one deletion cassette using primers Rdr1-f1 and Rdr1-r2. The resulting major PCR product was purified through ethanol precipitation and transformed into protoplasts of the wild-type strain HITO7711. Integration of the deletion cassette was confirmed by PCR using three primer sets (Rdr1-f3/Gen-chk-f1, Rdr1-f4/Rdr1-f4, and HPH-chk-r1/Rdr1-r3).

## Results

### Abnormal Ascospores Were Formed by Crossing Each Septin Null Mutant and the Wild-Type Strain

In order to find core septin genes in *B. maydis*, BLASTp searches were performed against the *B. maydis* C5 genome database on the NCBI website. The amino acid sequences of *S. cerevisiae* core septin proteins Cdc3 (YLR314C), Cdc10 (YCR002C), Cdc11 (YJR076C), and Cdc12 (YHR107C) were obtained from the DDBJ/EMBL/GenBank and used as queries. From the results of the BLASTp search using Cdc3, we found an encoding protein (COCHEDRAFT_1187161) with a high homology (52.32%) and designated this protein as Cdc3 in *B. maydis*. From the results of similar BLASTp searches using the remaining proteins, we found encoding proteins (COCHEDRAFT_101-8399, COCHEDRAFT_1019983, and COCHEDRAFT_1155150) with high homologies (58.17%, 46.53%, and 56.50%, respectively) to the *S. cerevisiae* Cdc10, Cdc11, and Cdc12, respectively. We designated these proteins as Cdc10, Cdc11, and Cdc12, respectively, in *B. maydis*.

To investigate the roles of core septin genes in *B. maydis*, we generated null mutants of each gene. We introduced deletion cassettes into the protoplasts of two wild-type strains, HITO7711 and MASHIKI2-2. The disruption of each gene was confirmed by PCR and the null mutants Δ*cdc3*, Δ*cdc10*, Δ*cdc11*, and Δ*cdc12* were obtained (**Supplementary Figure S1**). To observe the ascospore morphology, we performed cross tests. Each null mutant (*MAT1-2*) was crossed with the wild-type strain MASHIKI2-2 (*MAT1-1*). In *B. maydis*, eight filiform ascospores were formed in a single ascus by crossing the wild-type strains (**Figure 1**). Conversely, when each null mutant was crossed with the wild-type strain, all ascospores in a single ascus were not filiform but spindle shaped (**Figure 1**). Less than 5% of the asci contained normal ascospores (we counted over 1,000 ascospore-containing asci). These results show that core septins are involved in ascospore development. This phenomenon in which all ascospores from the same ascus display abnormal morphology is similar to the ascus dominance in *N. crassa*.

### The Ascus Dominance Resulting From *CDC10* Gene Disruption Was Caused by MSUD

**Figure 2:**
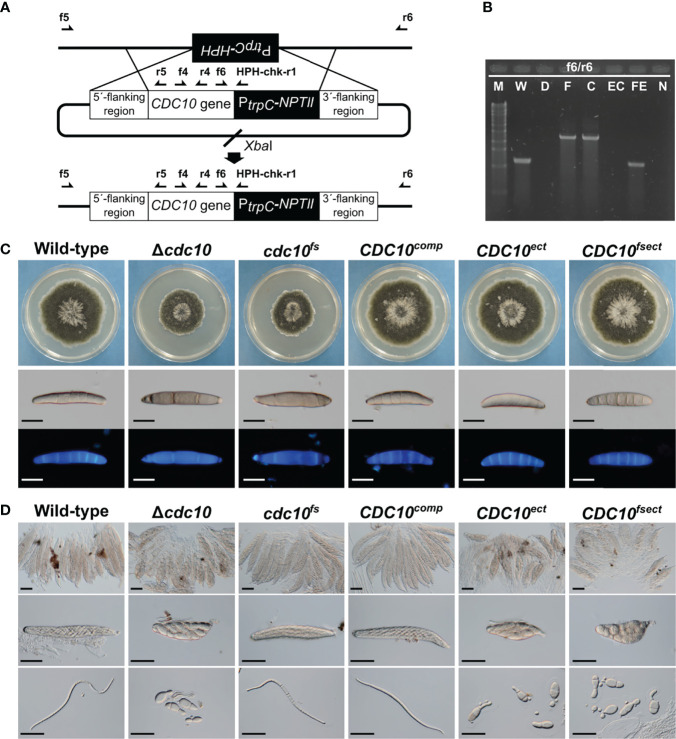
Generation and phenotype of mutants by the insertion of a *CDC10* sequence. **(A)** Schematic illustration of the introduction of linearized pZGCDC10c and the location of primers. The *CDC10^comp^* and the *cdc10^fs^* were generated by the insertion of each cassette into the Δ*cdc10* allele of the Δ*cdc10* strain. **(B)** PCR results for confirmation of the introduction of each *CDC10* cassette. Products were amplified by the primer set f6/r6 (W and WE: 1104 bp; F and C: 2321 bp). M, λ/*Sty*I digest; W, wild type; D, null mutant; F, *cdc10^fs^*; C, *CDC10^comp^*; EC, *CDC10^ect^*; FE, *CDC10^fsect^*; N, negative control (TE buffer). **(C)** Colonial growth and conidial morphology of the wild-type strain, the Δ*cdc10* strain, the *cdc10^fs^* strain, the *CDC10^comp^* strain, the *CDC10^ect^* strain, and the *CDC10^fsect^* strain. Each strain was cultured on CV8A for 7 days. Conidia of each strain were harvested from a colony of each strain on CV8A and stained with CFW. *Bars*: 50 µm. **(D)** Morphology of ascospores. Each strain was crossed with the wild-type strain on Sachs media. *Bars*: 50 µm.

The following attempts using the *CDC10* gene were made to clarify the involvement of MSUD in the ascus dominance of *B. maydis* core septins’ null mutants. The silencing by MSUD can be suppressed by pairing DNA in meiosis (Shiu et al., 2001). In order to ensure the pairing of *CDC10* alleles, we generated a new mutant allele with a single nucleotide deletion, causing a frameshift. The frameshift gene was inserted into the *CDC10* locus and the null allele was replaced *via* a double crossover event. The resulting transformant showed the expected resistance of geneticin, but the sensitivity of hygromycin and the correct integration was confirmed by PCR (**Figures 2A, B**, **Supplementary Figure S2**). The strain was designated as the *cdc10^fs^* strain. To confirm that the *cdc10^fs^* strain did not possess functional Cdc10, we tested the phenotypic aspects of the colony and conidia. The Δ*cdc10* strain showed a reduced growth rate compared with that of the wild-type strain (**Figure 2C**; **Table 1**). The reduced growth rate in the *cdc10^fs^* strain was similar to that in the Δ*cdc10* strain (**Figure 2C**; **Table 1**). The wild-type strain formed fusiform conidia, many of which possessed five to six septa (**Figure 2C**; **Table 1**). The conidia of the Δ*cdc10* strain had fewer septa compared with those of the wild-type strain. The numbers of septa in the conidia of the *cdc10^fs^* strain were similar to those in the Δ*cdc10* strain (**Figure 2C**; **Table 1**). These results indicated that *cdc10^fs^* was deficient in its functions.

**Table 1:** Vegetative growth and septation of *CDC10* mutants.

Strain	Colony diam. (mm^2^)	No. of septa/conidium
Wild-type	68.05 ± 0.4^a^	6.12 ± 0.2^a^
Δ*cdc10*	47.07 ± 0.5^b^	1.62 ± 0.1^b^
*cdc10^fs^*	42.89 ± 0.6^b^	1.56 ± 0.1^b^
*CDC10^comp^*	70.56 ± 1.3^a^	5.99 ± 0.2^a^
*CDC10^ect^*	68.77 ± 0.8^a^	6.03 ± 0.1^a^
*CDC10^fsect^*	70.60 ± 1.3^a^	6.26 ± 0.1^a^

Values represent the mean ± standard errors (colony diam. n=5; no. of septa/conidium n=100). Different letters indicate significant differences based on Tukey’s test (P < 0.05).

To investigate whether the pairing of the *CDC10* alleles in meiosis affects the morphology of ascospores, the *cdc10^fs^* strain was crossed with the wild-type strain. The resulting ascospores from crosses of the wild-type and Δ*cdc10* strains showed abnormal morphology. These ascospores were mostly spindle shaped with fewer cells, compared with a normal ascospore (**Figure 2D**). Interestingly, when the *cdc10^fs^* strain was crossed with the wild-type strain, the morphology of the ascospores was filiform shaped, similar to the normal ascospores from the cross between each wild-type strain, despite the lack of the *CDC10* gene in the *cdc10^fs^* strain (**Figure 2D**). The result showed that ascus dominance was suppressed by pairing the *CDC10* alleles.

We also generated a strain in which the frameshift cassette was ectopically integrated into a chromosome of the wild-type strain. The strain was designated as the *CDC10^fsect^* strain. The colony growth and conidial morphology of the *CDC10^fsect^* strain were not distinguishable from those of the wild-type strain (**Figure 2C**; **Table 1**). Conversely, as a result of crossing between the *CDC10^fsect^* and the wild-type strain, abnormal ascospores were formed even though these two strains possessed the wild-type *CDC10* gene in each genome (**Figure 2D**), suggesting that the ascus dominance occurred due to an unpaired *CDC10* frameshift sequence caused by the ectopic integration of this cassette. The result showed that the paired *CDC10* gene was also affected by ectopically introducing the nonfunctional *cdc10^fs^* sequence (*CDC10* frameshift sequence).

We also attempted to generate two reconstituted strains by reintroducing the wild-type *CDC10* gene into the Δ*cdc10* strain. The *CDC10^comp^* strain was generated by reintroducing the wild-type *CDC10* gene into the *CDC10* locus, replacing the null allele of the Δ*cdc10* strain. The *CDC10^ect^* strain was generated by ectopic integration of the same cassette into the Δ*cdc10* strain, retaining the null allele at the *CDC10* locus. These introductions of this cassette were confirmed by PCR (**Figure 2B**; **Supplementary Figure S2**). The colony growth and conidial morphology of the *CDC10^comp^* and *CDC10^ect^* strains were similar to those of the wild-type strain (**Figure 2C**; **Table 1**). These results showed that the inserted *CDC10* cassette provides the correct function to the strains introduced. Next, we crossed these strains with the wild-type strain. In the result of the cross between the *CDC10^comp^* strain and the wild-type strain, the defect in ascospores was recovered, resulting in the production of filiform ascospores (**Figure 2D**). Conversely, when the *CDC10^ect^* strain was crossed with the wild-type strain, abnormal ascospores were formed, similar to those from the cross between the Δ*cdc10* strain and the wild-type strain (**Figure 2D**). These results indicated that even if a functional *CDC10* gene is inserted, ascus dominance results from ectopic integration. Based on the above results, in *B. maydis*, ascus dominance is thought to be triggered by an unpaired *CDC10* sequence, suggesting that this phenomenon is caused by MSUD.

### The *RDR1* Gene Encoding the MSUD-Related RdRP in *B. maydis* Was Identified

**Figure 3:**
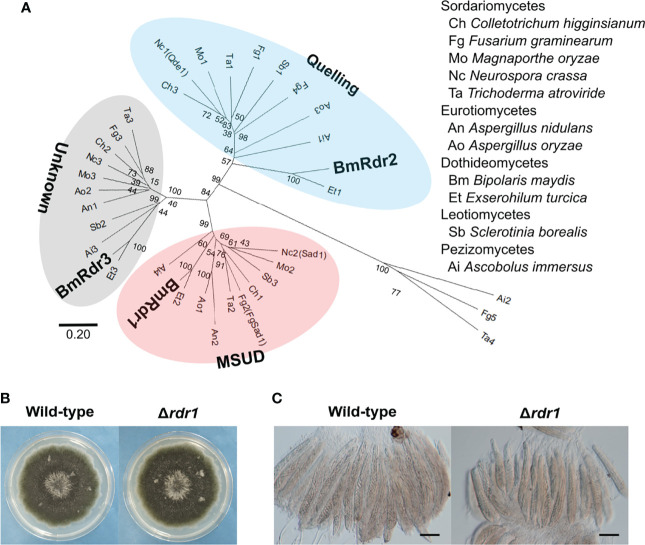
Identification of the MSUD-related RdRP and generation of the *RDR1* disruption mutant in *B. maydis*. **(A)** Phylogenetic analysis of the RdRP protein sequence. A neighbor-joining tree was constructed based on the conserved domain of the RdRP. The locus tags of proteins used in this analysis are listed in **Supplementary Table S2**. The main three groups—quelling, MSUD, and unknown—are divided into different color zones. **(B)** Colonial morphology of the Δ*rdr1* strain. The wild-type strain and the Δ*rdr1* strain were cultured on CV8A for 7 days. **(C)** Asci and ascospores from the cross between the Δ*rdr1* strain and the wild-type strain. The Δ*rdr1* strain was crossed with the wild-type strain. After 4 weeks, resulting pseudothecia were harvested and dissected to observe the asci and ascospores. *Bars*: 50 µm.

MSUD is known to be an RNAi-related phenomenon during meiosis (Shiu et al., 2001). The phenomenon is mainly regulated by three factors: the RdRP (Shiu et al., 2001), the Argonaute-like protein (Lee et al., 2003), and the Dicer-like protein (Alexander et al., 2008). Among these, the lack of an RdRP suppresses MSUD (Shiu et al., 2001). To find RdRPs in *B. maydis*, we carried out a BLASTp search at the NCBI website. The RdRP domain (707–1083 aa) in *N. crassa* Qde1 (NCU07534) was used as a query, as described in a previous publication (Nakayashiki et al., 2006). As a result of the BLAST search, three proteins containing the RdRP domain were found in the *B. maydis* C5 genome database on the NCBI website. To predict the RdRP function related to MSUD, we performed a phylogenetic analysis, which showed that the RdRPs were divided into three groups (**Figure 3**). Sad-1 proteins encode the RdRP in *N. crassa* and *F. graminearum*, which are essential for MSUD in both fungi (Shiu et al., 2001; Son et al., 2011). The RdRP (COCHEDRAFT_1138647) in *B. maydis* belonged to the same group as these proteins. Therefore, this RdRP was thought to function with relation to MSUD in *B. maydis* and was designated as Rdr1.

To investigate the functions of the *RDR1* gene in *B. maydis*, we generated null mutants of the gene and obtained transformants that showed the expected resistance to geneticin. Subsequently, these transformants were confirmed by PCR and characterized as the *RDR1* null mutant (Δ*rdr1*; **Supplementary Figure S3**). The Δ*rdr1* strain showed colonial morphology in CV8A similar to that of the wild-type strain (**Figure 3B**). In the results of crossing the Δ*rdr1* strain and the wild-type strain, filiform ascospores were formed, as in the case of the wild-type (**Figure 3C**). These results show that deletion of the *RDR1* gene has no effect on colony or ascospore development.

### Ascus Dominance by the Unpaired *CDC10* Allele Was Suppressed by the Δ*rdr1* Strain

**Figure 4:**
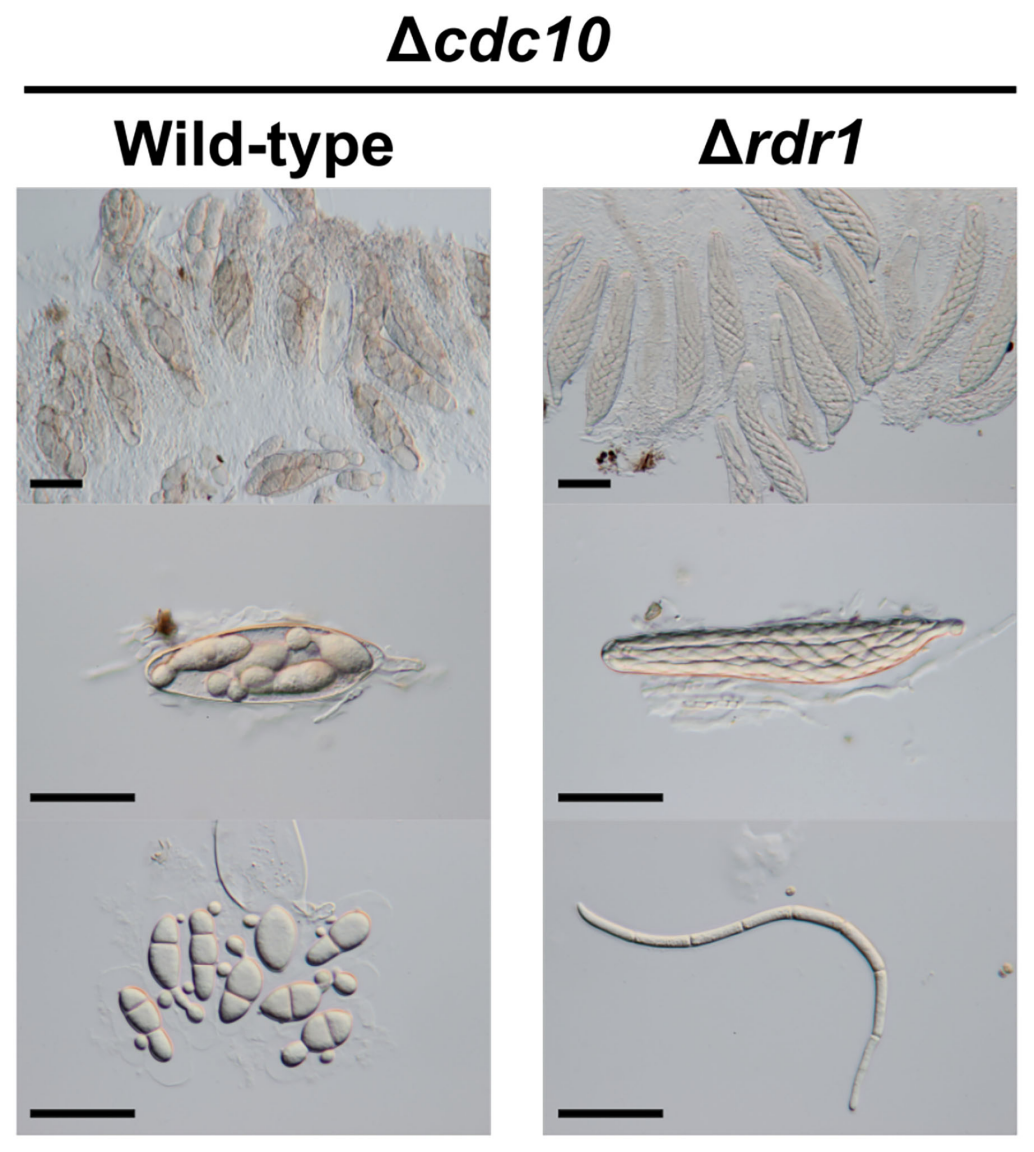
Asci and ascospores from the cross between the Δ*rdr1* strain and the Δ*cdc10* strain. The Δ*rdr1* strain was crossed with the Δ*cdc10* strain. After 4 weeks, resulting pseudothecia were harvested and dissected to observe the asci and ascospores. *Bars*: 50 µm.

To investigate whether MSUD was suppressed by the absence of the Rdr1 in *B. maydis*, we performed a cross test of the Δ*rdr1* strain and the Δ*cdc10* strain. The Δ*cdc10* strain formed abnormal ascospores in the cross with the wild-type strain (**Figure 4**). Conversely, the result from the cross between the Δ*rdr1* strain and the Δ*cdc10* strain showed that the morphology of the ascospores was filiform, as in the case of ascospores obtained from the cross between the wild-type strains, suggesting that ascus dominance did not occur in this combination (**Figure 4**). The result shows that disruption of the *Rdr1* gene suppressed MSUD in *B. maydis*, suggesting that Rdr1 had a major function in MSUD in *B. maydis* as well as in *F. graminearum* and *N. crassa*.

### The Ascus Dominance by Each Unpaired Core Septin Allele Was Also Suppressed by the Δ*rdr1* Strain

**Figure 5:**
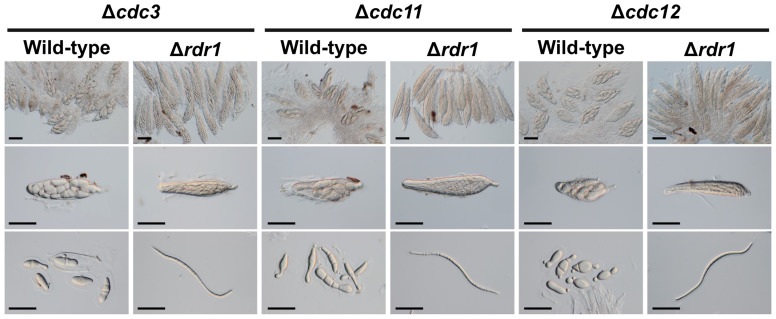
Asci and ascospores from crosses between the Δ*rdr1* strain and null mutants of the *CDC3*, *CDC11*, and *CDC12* genes. The Δ*rdr1* strain was crossed with the null mutants of septins. After 4 weeks, resulting pseudothecia were harvested and dissected to observe the asci and ascospores. *Bars*: 50 µm.

Other disruption mutants of core septin genes, the Δ*cdc3*, Δ*cdc11*, and Δ*cdc12* strains, also formed abnormal ascospores in crosses with the wild-type strain (**Figures 1**, **5**). To investigate whether the ascus dominance by these null mutants was also caused by MSUD, these null mutants were crossed with the Δ*rdr1* strain. The results of hybridization tests showed that the ascospores from each cross were filiform in shape. These results indicated that three core septin genes were also affected by MSUD when each null mutant was crossed with the wild-type strain. MSUD is known to function from meiosis to ascospore formation (Son et al., 2011). If a gene was affected by MSUD, that gene became a necessary factor for karyogamy to ascospore formation. Thus, these results suggest that core septin genes in *B. maydis* are important for the karyogamy to ascospore formation.

## Discussion

Gene disruption methods are often used to analyze the function of the genes in fungi. Morphological changes due to lack of the genes are an important source for the estimate of gene functions. Occasionally, however, the morphological phenotype of the ascospore does not correspond with the genotype that the ascospores possess. In this study, null mutants of four core septins (Δ*cdc3*, Δ*cdc10*, Δ*cdc11*, and Δ*cdc12* strains) were generated in *B. maydis*. When each null mutant was crossed with the wild-type strain, the eight ascospores derived from a single ascus showed an entirely abnormal morphology (**Figure 1**). From this result, we noticed that the abnormal morphology of the ascospores appears as a dominant phenotype regardless of the genotype of the ascospores. We also thought that the phenomenon was remarkably similar to the ascus dominance in *N. crassa*.

Ascus dominance is a phenomenon in which a recessive trait appears predominantly in the ascospores within a single ascus, regardless of the genotype of the ascospore. This phenomenon was observed in a cross between the wild-type strain and a null mutant of the *Asm-1* gene, which encodes the APSES transcription factor in *N. crassa* (Aramayo et al., 1996; Shiu et al., 2001). In *F. graminearum*, when null mutants of the *Roa* gene which encodes a ketopantoate reductase domain-containing protein, and the *FgAma1* gene which encodes a meiosis coactivator of the APC/C (anaphase-promoting complex/cyclosome), are crossed with the wild-type strain, ascus dominance appeared in the formed ascospores (Min et al., 2010; Son et al., 2011; Hao et al., 2019). The ascus dominance observed in these cases is thought to be caused by MSUD. MSUD is posttranscriptional RNAi-related regulation that occurs specifically in meiosis and is presumed to silence a gene that does not pair properly in chromosomes during meiosis. When a null mutant is crossed with the wild-type strain, the null allele fails to pair with the wild-type allele and the wild-type gene is silenced during meiosis. In *N. crassa* and *F. graminearum*, since the ascus dominance is mainly caused by MSUD, the suppression or induction of ascus dominance was utilized to demonstrate MSUD. In general, two methods are used, as described below. First, a homologous sequence (single nucleotide deletion or frameshift mutations) was introduced to the null allele to ensure pairing of the genes during meiosis, resulting in the suppression of ascus dominance (Aramayo and Metzenberg, 1996; Hao et al., 2019). Next, the mutant carrying the ectopic copy of a gene (functional or nonfunctional) was generated and crossed with the wild-type strain. Since the ectopic copy is unpaired, ascus dominance occured (Aramayo and Metzenberg, 1996; Son et al., 2011). In this study, to confirm whether the ascus dominance, as a result of crossing the Δ*cdc10* strain with the wild-type strain in *B. maydis*, is caused by MSUD, we performed cross tests using the strategies described above. In this study, the *cdc10^fs^* strain was generated by introducing a sequence comparable to that of the wild-type strain (frameshift sequence of the *CDC10* gene) to the null allele in the Δ*cdc10* strain. When the *cdc10^fs^* strain was crossed with the wild-type strain, ascus dominance did not occur (**Figure 2D**). Next, we generated two mutants: the *CDC10^ect^* strain carrying the functional ectopic *CDC10* gene and no endogenous gene and the *CDC10^fsect^* strain carrying the nonfunctional ectopic *CDC10* gene and the endogenous gene. When the two mutants were crossed with the wild-type strain, ascus dominance occurred (**Figure 2D**). These results were similar to those in *N. crassa* and *F. graminearum*. Hence, MSUD caused the ascus dominance observed in the ascospores derived from the cross between the Δ*cdc10* and wild-type strains and was also functional in *B. maydis*.

MSUD is regulated by three RNAi components (the RdRP, the Argonaute-like protein, and the Dicer-like protein; Shiu et al., 2001; Lee et al., 2003; Alexander et al., 2008, respectively). Among them, it is thought that the MSUD-related RdRP recognizes the aberrant RNA produced from the unpaired gene and synthesizes double-stranded RNA (Dang et al., 2011). In *N. crassa* and *F. graminearum*, the *Sad-1* gene encoding the MSUD-specific RdRP is essential for MSUD (Shiu et al., 2001; Son et al., 2011). In this study, the *RDR1* gene in *B. maydis* was identified as a *Sad-1* orthologue by searching the genome databases and conducting a subsequent phylogenetic analysis (**Figure 3A**). To investigate whether Rdr1 is the main regulator of MSUD in *B. maydis*, the Δ*rdr1* strain was made. In the result from the cross between the Δ*rdr1* strain and the Δ*cdc10* strain, ascus dominance was expectedly suppressed (**Figure 4**). These results showed that the *RDR1* gene in *B. maydis* is essential for MSUD, as in the case of *N. crassa* and *F. graminearum*. The filamentous ascomycetes possess several RdRPs (**Supplementary Table S2**). The RdRPs are divided into at least three groups: quelling (silencing in the vegetative phase), MSUD, and unknown (Campo et al., 2016). In this study, the phylogenetic tree showed a consistent result (**Figure 3A**). Interestingly, all filamentous ascomycetes used in the phylogenetic analysis possessed RdRP in the group of MSUD (**Figure 3A**). It has been suggested that RNAi components involved in the MSUD pathway arose only in ascomycetes (Nakayashiki et al., 2006). However, it is not clear whether MSUD is functional among these fungi. To date, MSUD has been reported only in *N. crassa* and *F. graminearum*, which belong to the Sordariomycete class. In addition to Sordariomycetes, in this study, we showed that MSUD is functional in *B. maydis*, belonging to the class Dothideomycetes, another class of ascomycetes. The combined results from the phylogenetic analysis and the experimental evidence of MSUD in *B. maydis* suggest that MSUD is functional in a wide range of filamentous ascomycetes with MSUD-related RNAi components.

Septins (GTP-binding proteins), are widely conserved in eukaryotes, except in plants. Core septins play important roles for morphological development and pathogenicity in fungi (Berepiki and Read, 2013; Gupta et al., 2015; Zhang et al., 2020). In this study, the silencing of core septin genes by MSUD led to the formation of abnormal ascospores (**Figures 4**, **5**), The formed abnormal ascospores are spindle shaped and significantly shorter than normal ascospores (**Figures 4**, **5**), indicating that septins are involved in the elongation of ascospore membranes. However, in the cross of the *cdc10^fs^* strain and the wild-type strain, all ascospores developed normally and showed filiform. The similar result was obtained from the cross of the Δ*rdr1* strain and the Δ*cdc10* strain. These will imply the quantity of septin generated from the haploid gene is sufficient for ascospore development in the ascus.

Core septins formed higher-order structures in hyphae and appressoria in filamentous fungi (Berepiki and Read, 2013; Gupta et al., 2015). Thus, it is necessary to investigate the structures of core septins in ascosporogenesis. In *N. crassa*, when the mutant carrying the fluorescent protein gene was crossed with the wild-type strain, fluorescence could not be observed in the ascus due to silencing of the gene by MSUD (Freitag et al., 2004). However, fluorescence could be observed when MSUD was suppressed (Freitag et al., 2004; Shiu et al., 2006). These results suggested that the Δ*rdr1* strain can be utilized as a tool to analyze the localization of core septins using fluorescent proteins in the ascosporogenesis of *B. maydis*. Research into observing septin within asci is already in progress.

## Data Availability Statement

The original contributions presented in the study are included in the article/**Supplementary Material**. Further inquiries can be directed to the corresponding author.

## Author Contributions

KT, AY, and CT conceived and designed the experiments. KT and YK constructed fungal mutants. KT performed most experiments and analyzed the data. All authors contributed to the article and approved the submitted version.

## Funding

Part of this work was supported financially by Grants-in-Aid for Scientific Research (C) [JP19K06052] from the Japan Society for the Promotion of Science to CT.

## Conflict of Interest

The authors declare that the research was conducted in the absence of any commercial or financial relationships that could be construed as a potential conflict of interest.

## Publisher’s Note

All claims expressed in this article are solely those of the authors and do not necessarily represent those of their affiliated organizations, or those of the publisher, the editors and the reviewers. Any product that may be evaluated in this article, or claim that may be made by its manufacturer, is not guaranteed or endorsed by the publisher.

## References

[B1] Alexander W. G. Raju N. B. Xiao H. Hammond T. M. Perdue T. D. Metzenberg R. L.. (2008). DCL-1 Colocalizes With Other Components of the MSUD Machinery and Is Required for Silencing. Fungal Genet. Biol. 45, 719–727. doi: 10.1016/j.fgb.2007.10.006 18036854

[B2] Aramayo R. Metzenberg R. L. (1996). Meiotic Transvection in Fungi. Cell 86, 103–113. doi: 10.1016/S0092-8674(00)80081-1 8689677

[B3] Aramayo R. Peleg Y. Addison R. Metzenberg R. (1996). *Asm-1+*, a *Neurospora Crassa* Gene Related to Transcriptional Regulators of Fungal Development. Genetics 144, 991–1003. doi: 10.1093/genetics/144.3.991 8913744 PMC1207638

[B4] Berepiki A. Read N. D. (2013). Septins are Important for Cell Polarity, Septation and Asexual Spore Formation in *Neurospora Crassa* and Show Different Patterns of Localisation at Germ Tube Tips. PloS One 8, e63843. doi: 10.1371/journal.pone.0063843 23691103 PMC3653863

[B5] Campo S. Gilbert K. B. Carrington J. C. (2016). Small RNA-Based Antiviral Defense in the Phytopathogenic Fungus *Colletotrichum Higginsianum* . PloS Pathog. 12, 1–36. doi: 10.1371/journal.ppat.1005640 PMC4890784 27253323

[B6] Carroll A. M. Sweigard J. A. Valent B. (1994). Improved Vectors for Selecting Resistance to Hygromycin. Fungal Genet. Rep. 41, 5. doi: 10.4148/1941-4765.1367

[B7] Dang Y. Yang Q. Xue Z. Liu Y. (2011). RNA Interference in Fungi: Pathways, Functions, and Applications. Eukaryot. Cell 10, 1148–1155. doi: 10.1128/EC.05109-11 21724934 PMC3187057

[B8] Davidow L. S. Goetsch L. Byers B. (1980). Preferential Occurrence of Nonsister Spores in Two-Spored Asci of *Saccharomyces Cerevisiae*: Evidence for Regulation of Spore-Wall Formation by the Spindle Pole Body. Genetics 94, 581–595. doi: 10.1093/genetics/94.3.581 17249010 PMC1214161

[B600] Edgar R. C. (2004). MUSCLE: Multiple Sequence Alignment With High Accuracy and High Throughput., Nucleic Acids Res. 32 (5), 1792–1797. doi: 10.1093/nar/gkh340 15034147 PMC390337

[B9] Erwin D. C. McCormick W. H. (1971). Germination of Oospores Produced by *Phytophthora Megasperma* Var. *Sojae* . Mycologia 63, 972–977. doi: 10.2307/3757899

[B10] Finn R. D. Coggill P. Eberhardt R. Y. Eddy S. R. Mistry J. Mitchell A. L.. (2016). The Pfam Protein Families Database: Towards a More Sustainable Future. Nucleic Acids Res. 44, D279–D285. doi: 10.1093/nar/gkv1344 26673716 PMC4702930

[B11] Freitag M. Hickey P. C. Raju N. B. Selker E. U. Read N. D. (2004). GFP as a Tool to Analyze the Organization, Dynamics and Function of Nuclei and Microtubules in *Neurospora Crassa* . Fungal Genet. Biol. 41, 897–910. doi: 10.1016/j.fgb.2004.06.008 15341912

[B12] Gupta Y. K. Dagdas Y. F. Martinez-Rocha A. L. Kershaw M. J. Littlejohn G. R. Ryder L. S.. (2015). Septin-Dependent Assembly of the Exocyst is Essential for Plant Infection by *Magnaporthe Oryzae* . Plant Cell 27, 3277–3289. doi: 10.1105/tpc.15.00552 26566920 PMC4682301

[B13] Hao C. Yin J. Sun M. Wang Q. Liang J. Bian Z.. (2019). The Meiosis-Specific APC Activator *FgAMA1* Is Dispensable for Meiosis But Important for Ascosporogenesis in *Fusarium Graminearum* . Mol. Microbiol. 111, 1245–1262. doi: 10.1111/mmi.14219 30746783

[B14] Izumitsu K. Hatoh K. Sumita T. Kitade Y. Morita A. Gafur A.. (2012). Rapid and Simple Preparation of Mushroom DNA Directly From Colonies and Fruiting Bodies for PCR. Mycoscience 53, 396–401. doi: 10.1007/s10267-012-0182-3

[B15] Izumitsu K. Yoshimi A. Kubo D. Morita A. Saitoh Y. Tanaka C. (2009). The MAPKK Kinase ChSte11 Regulates Sexual/Asexual Development, Melanization, Pathogenicity, and Adaptation to Oxidative Stress in *Cochliobolus Heterostrophus* . Curr. Genet. 55, 439–448. doi: 10.1007/s00294-009-0257-7 19547975

[B16] Kitade Y. Sumita T. Izumitsu K. Tanaka C. (2019). Cla4 PAK-Like Kinase Is Required for Pathogenesis, Asexual/Sexual Development and Polarized Growth in *Bipolaris Maydis* . Curr. Genet. 65, 1229–1242. doi: 10.1007/s00294-019-00977-9 31028454

[B17] Kumar S. Stecher G. Li M. Knyaz C. Tamura K. (2018). MEGA X: Molecular Evolutionary Genetics Analysis Across Computing Platforms. Mol. Biol. Evol. 35, 1547–1549. doi: 10.1093/molbev/msy096 29722887 PMC5967553

[B18] Lee D. W. Pratt R. J. McLaughlin M. Aramayo R. (2003). An Argonaute-Like Protein is Required for Meiotic Silencing. Genetics 164, 821–828. doi: 10.1093/genetics/164.2.821 12807800 PMC1462569

[B19] Min K. Lee J. Kim J. C. Kim S. G. Kim Y. H. Vogel S.. (2010). A Novel Gene, ROA, Is Required for Normal Morphogenesis and Discharge of Ascospores in *Gibberella Zeae* . Eukaryot. Cell 9, 1495–1503. doi: 10.1128/EC.00083-10 20802018 PMC2950417

[B21] Nakayashiki H. Kadotani N. Mayama S. (2006). Evolution and Diversification of RNA Silencing Proteins in Fungi. J. Mol. Evol. 63, 127–135. doi: 10.1007/s00239-005-0257-2 16786437

[B22] Raju N. B. (1994). Ascomycete Spore Killers: Chromosomal Elements That Distort Genetic Ratios Among the Products of Meiosis. Mycologia 86, 461–473. doi: 10.2307/3760737

[B23] Raju N. B. (2008). Meiosis and Ascospore Development in *Cochliobolus Heterostrophus* . Fungal Genet. Biol. 45, 554–564. doi: 10.1016/j.fgb.2007.08.007 17931917

[B24] Ribeiro O. K. (1978). A Source Book of Genus Phytophthora. Vaduz, Liechtenstein: J. Cramer.

[B25] Sambrook J. Fritsch E. F. Maniatis T. (1989). Molecular Cloning: A Laboratory Manual (2nd Ed.) (Cold Spring Harbor Laboratory, Cold Spring Harbor, NY).

[B26] Shiu P. K. T. Raju N. B. Zickler D. Metzenberg R. L. (2001). Meiotic Silencing by Unpaired DNA. Cell 107, 905–916. doi: 10.1016/S0092-8674(01)00609-2 11779466

[B27] Shiu P. K. T. Zickler D. Raju N. B. Ruprich-Robert G. Metzenberg R. L. (2006). SAD-2 Is Required for Meiotic Silencing by Unpaired DNA and Perinuclear Localization of SAD-1 RNA-Directed RNA Polymerase. Proc. Natl. Acad. Sci. U. S. A. 103, 2243–2248. doi: 10.1073/pnas.0508896103 16461906 PMC1413707

[B28] Son H. Min K. Lee J. Raju N. B. Lee Y. W. (2011). Meiotic Silencing in the Homothallic Fungus *Gibberella Zeae* . Fungal Biol. 115, 1290–1302. doi: 10.1016/j.funbio.2011.09.006 22115448

[B29] Sumita T. Izumitsu K. Tanaka C. (2017). Characterization of the Autophagy-Related Gene *BmATG8* in *Bipolaris Maydis* . Fungal Biol. 121, 785–797. doi: 10.1016/j.funbio.2017.05.008 28800850

[B30] Szewczyk E. Nayak T. Oakley C. E. Edgerton H. Xiong Y. Taheri-Talesh N.. (2006). Fusion PCR and Gene Targeting in *Aspergillus Nidulans* . Nat. Protoc. 1, 3111–3120. doi: 10.1038/nprot.2006.405 17406574

[B31] Tanaka C. Kubo Y. Tsuda M. (1991). Genetic Analysis and Characterization of *Cochlioborus Heterostrophus* Colour Mutants. Mycol. Res. 95, 49–56. doi: 10.1016/S0953-7562(09)81360-9

[B32] Turgeon B. G. (1998). Application of Mating Type Gene Technology to Problems in Fungal Biology. Annu. Rev. Phytopathol. 36, 115–137. doi: 10.1146/annurev.phyto.36.1.115 15012495

[B33] Zhang X. González J. B. Turgeon B. G. (2020). Septins Are Required for Reproductive Propagule Development and Virulence of the Maize Pathogen *Cochliobolus Heterostrophus* . Fungal Genet. Biol. 135, 103291. doi: 10.1016/j.fgb.2019.103291 31698077

