# Supplementary material for: Meiotic Silencing in Dothideomycetous Bipolaris maydis
Source: Front Fungal Biol. 2022 Jun 27;3:931888. doi: 10.3389/ffunb.2022.931888 (PMC10512333; doi:10.3389/ffunb.2022.931888)
Supplement: Supplementary file 1 [file Table_1.docx]

Supplementary Material

**Meiotic silencing in Dothideomycetous *Bipolaris maydis***

**Kenya Tsuji, Yuki Kitade, Akira Yoshimi, and Chihiro Tanaka***

*** Correspondence:** Chihiro Tanaka: tanaka.chihiro.6a@kyoto-u.ac.jp

**Supplementary Table S1. Primers used in this study**

| Name | Nucleotide sequence (5′***–***3′) |
| --- | --- |
| Cdc3-f1 | CTAGTGCACATTGCTTTGGCGTTGCTC |
| Cdc3-r1 | CTGAGCAAACTGGCCTCAGGCATTTGAGAAGCACCAGGGGAAGCAGAAAAGCAACCTTGTG |
| Cdc3-f2 | GATCAAAAAGTGCTCATCATTGGAAAACGTTCTTCGTTGCCCCTGGCCTTCTCACTTCTC |
| Cdc3-r2 | GCAACAATTCTGGGTTGTGCAGGTG |
| Cdc3-f3 | CCGGCACTGTCTGCGAATGGTGAC |
| Cdc3-r3 | GTGGGCAGTAACCCAGGCTTGGTC |
| Cdc-f4 | TTTCGCCAACCTGCCCAACCAGTG |
| Cdc3-r4 | GGCCTTGGCAATGACGGGAATCAG |
| Cdc10-f1 | GGCAGAGAATGTGCAGTCGCTTCAC |
| Cdc10-r1 | CTGAGCAAACTGGCCTCAGGCATTTGAGAAGCACATGTTATGGGGGAGCGAGACCTTGTG |
| Cdc10-f2 | GATCAAAAAGTGCTCATCATTGGAAAACGTTCTTCGGTCGCGTCGATAGCAATACAGGCAG |
| Cdc10-r2 | CGGTATCATCCACAGATGCAAGGAG |
| Cdc10-f3 | CAATGCCACGAGCGTGTCCTGAGAC |
| Cdc10-r3 | CGTGTTACCCAGAACGGTTAGGCTC |
| Cdc10-f4 | CGTTGTTCCCGTCATTGCCAAGAGTG |
| Cdc10-r4 | GTCGTCTCGATCAGGTCTTGCAAG |
| Cdc11-f1 | TAGCCGATTCTGGCAAGCGTGACTG |
| Cdc11-r1 | CTGAGCAAACTGGCCTCAGGCATTTGAGAAGCACGTCTTGAATGGAGTCGTGTGCACAC |
| Cdc11-f2 | GATCAAAAAGTGCTCATCATTGGAAAACGTTCTTCGGGCTTGGACGGGCAAAAAAGAAGAG |
| Cdc11-r2 | TTTCCCTGGCGTGCACAAGGTAGTG |
| Cdc11-f3 | CAGCTCACCTTGTCATCTGCATCCAC |

**Supplementary Table S1 (continued)**

| Cdc11-r3 | GGCGGTGAGGAGTATTATGCCGTG |
| --- | --- |
| Cdc11-f4 | GCTACCCTGCCATCAATTCCAGTCAC |
| Cdc11-r4 | GCCAGTAGGCGTGATGAAGTATAGCAG |
| Cdc12-f1 | CAGGTTGCCTCTGCTGTAGACAGTG |
| Cdc12-r1 | CTGAGCAAACTGGCCTCAGGCATTTGAGAAGCACTACGGGGAAAGGGTCTACAGGGAC |
| Cdc12-f2 | GATCAAAAAGTGCTCATCATTGGAAAACGTTCTTCGTGGCAACGATTGTCGAGATTCTTCCTC |
| Cdc12-r2 | AGGCGCATTTGAAACAGCTCGAAGAC |
| Cdc12-f3 | GAAGACAGTCACGCGATTGCCTACAG |
| Cdc12-r3 | CAGACCAGATTCTCTCCTCGTGCAC |
| Cdc12-f4 | CCATCTGATACCTCTAGGTTGCTGGAG |
| Cdc12-r4 | GAGCGGTCAGCATGTGTTGGCAG |
| pCB1004-f1 | GTGCTTCTCAAATGCCTGAG |
| pCB1004-r1 | CGAAGAACGTTTTCCAATG |
| HPH-chk-f1 | TGTAGAAGTACTCGCCGATAGTGG |
| HPH-chk-r1 | GTTGCCTAAATGAACCATCTTGTC |
| Cdc10-PG-f1 | GCTGGAATTCTGCAGATAGTTTGGTAACCTTGGATCGCTTGGATCGATCTTTAACAAGTGTC |
| Cdc10-PGm-r1 | TGGTTCTGGTACGCGGCAGCCATTG |
| Cdc10-PGm-f1 | CAATGGCTGCCGCGTACCAGAACCAGTCGCAGCCCATCTTCCCTGACAGCTATG |
| Cdc10-PG-r1 | AACGTTCTTCGGATATCAGTGAGGAAAGAGCAAGGGAGTATGG |
| Cdc10r-f1 | CACCGGCTTCGGACCAGGCCATTG |
| Cdc10r-r1 | GAGCCAAGAGAGAATACTACTGCTTCC |
| Cdc10-f5 | TCGGCATCAGCGAAAGGAAGTGAATTG |
| Cdc10-r5 | CCTATTAGTGGGCGTGTACACAAGAC |
| Cdc10-f6 | TCGGCATCAGCGAAAGGAAGTGAATTG |
| Cdc10-r6 | CCAGAGGACGGAGGAGCTGATAAG |
| Rdr1-f1 | GACTCTCGCATCTACTACAAAGCTAGC |

**Supplementary Table S1 (continued)**

| Rdr1-r1 | CCTCAGGCATTTGAGAAGCACGATTTTGCTCGGTTAGTCTTGGAATGG |
| --- | --- |
| Rdr1-f2 | CTCATCATTGGAAAACGTTCTTCGGAGACCTAGAGCGAGTGGGTAAGC |
| Rdr-r2 | GGAAGTCGTCGATTTTGCCCTTGTTGC |
| Rdr1-f3 | CTTACAAGGGGTTTCCTGTTCTTTCG |
| Rdr1-r3 | CGTTGCGTGCGGTAAGATGATGTTTG |
| Rdr1-f4 | GCAAAGGATGGCGTTCAGGTGATGC |
| Rdr1-r4 | GATGTTTACGTACCAATGTCGATGATAGC |
| Gen-r1 | GTGCTTCTCAAATGCCTGAGGCCAGTTTGCTCAGTCAGAAGAACTCGTCAAGAAGG |
| Gen-chk-f1 | CGTGATATTGCTGAAGAGCTTGG |

Underlines show the overlapping sequences for PCR fusion.

**Supplementary Table S2. Candidate RdRP used in phylogenetic analysis in this study**

|  |  | Candidate RdRP | | | | |
| --- | --- | --- | --- | --- | --- | --- |
| Class | Fungi | Locus tag | | | | |
| Sordariomycetes | *Colletotrichum higginsianum* | CH063_02767 | CH063_05776 | CH063_08349 |  |  |
|  | *Fusarium graminearum* | FGSG_06504 | FGSG_08716 | FGSG_01582 | FGSG_04619 | FGSG_09076 |
|  | *Magnaporthe oryzae* | MGG_13453 | MGG_02748 | MGG_06205 |  |  |
|  | *Neurospora crassa* | NCU02178 | NCU07534.1 | NCU08435.1 |  |  |
|  | *Trichoderma atroviride* | TRIATDRAFT_321718 | TRIATDRAFT_225118 | TRIATDRAFT_317554 | TRIATDRAFT_294361 |  |
| Eurotiomycetes | *Aspergillus nidulans* | AN2717.2 | AN4790.2 |  |  |  |
|  | *Aspergillus oryzae* | AO090020000318 | AO090103000121 | AO090020000563 |  |  |
| Dothideomycetes | *Bipolaris maydis* | COCHEDRAFT_1138647 | COCHEDRAFT_1201293 | COCHEDRAFT_106396 |  |  |
|  | *Exserohilum turcica* | SETTUDRAFT_137938 | SETTUDRAFT_99344 | SETTUDRAFT_146609 |  |  |
| Leotiomycetes | *Sclerotinia borealis* | SBOR_5459 | SBOR_9633 | SBOR_2395 |  |  |
| Pezizomycetes | *Ascobolus immersus* | BJ508DRAFT_40060 | BJ508DRAFT_414356 | BJ508DRAFT_66649 | BJ508DRAFT_363736 |  |
