# Supplementary material for: Meiotic Silencing in Dothideomycetous Bipolaris maydis
Source: Front Fungal Biol. 2022 Jun 27;3:931888. doi: 10.3389/ffunb.2022.931888 (PMC10512333; doi:10.3389/ffunb.2022.931888)
Supplement: Supplementary file 2 [file Image_1.pdf]

Supplementary Material

Meiotic silencing in Dothideomycetous *Bipolaris maydis*

Kenya Tsuji, Yuki Kitade, Akira Yoshimi, and Chihiro Tanaka*

*** Correspondence:** Chihiro Tanaka: tanaka.chihiro.6a@kyoto-u.ac.jp

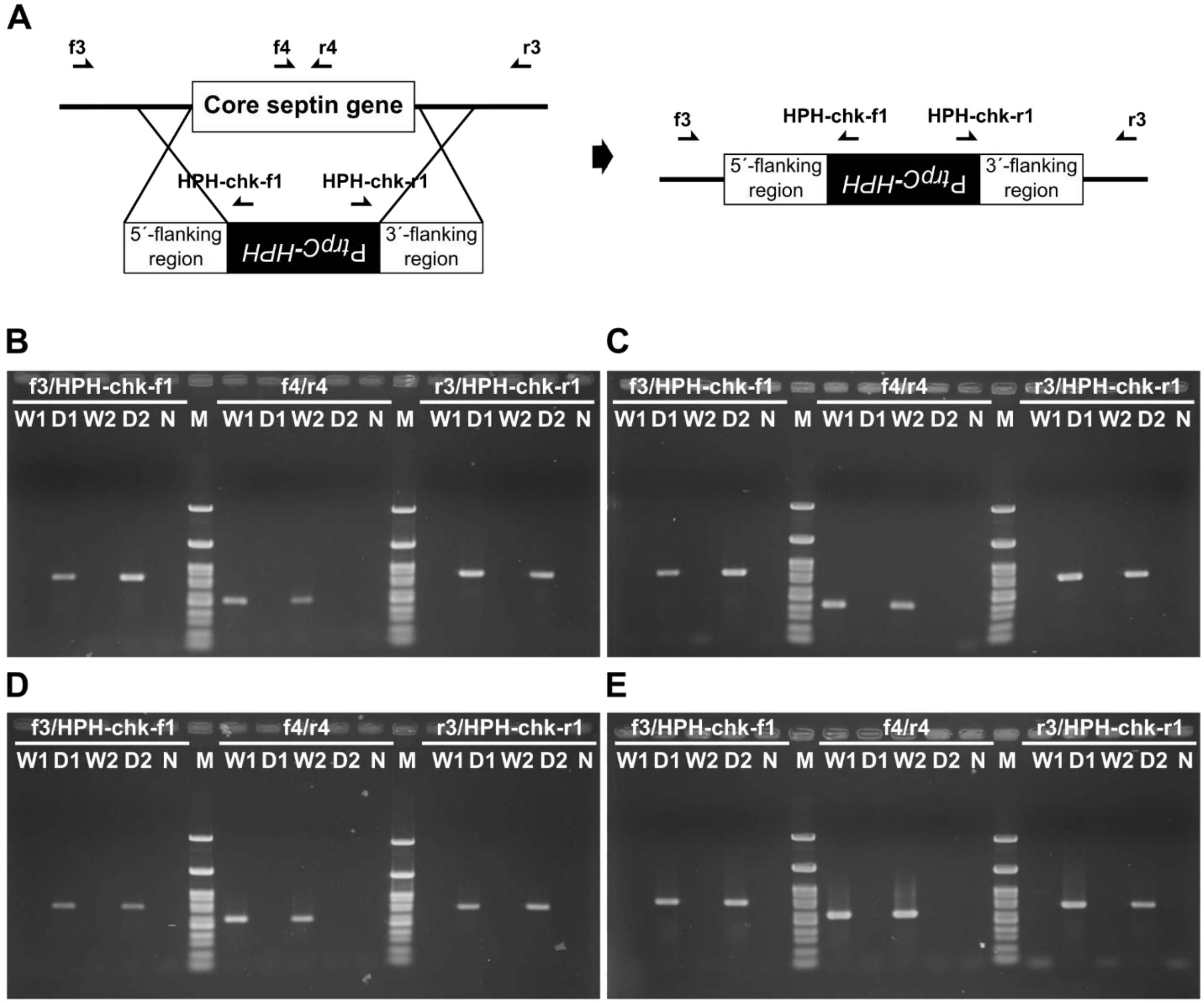

Supplementary Figure S1. Disruption of the *CDC3*, *CDC10*, *CDC11*, and *CDC12* genes in *B. maydis*. (A) Schematic illustration of the disruption of three genes and the location of primers. (B) PCR results for the confirmation of *CDC3* gene disruption. Left: Products amplified by the primer set f3/HPH-chk-f1 (838 bp). Middle: Products amplified by the primer set f4/r4 (550 bp). Right: Products amplified by the primer set HPH-chk-r1/r3 (876 bp). (C) PCR results for the confirmation of *CDC10* gene disruption. Left: Products amplified by the primer set f3/HPH-chk-f1 (843 bp). Middle: Products amplified by the primer set f4/r4 (453 bp). Right: Products amplified by the primer set HPH-chk-r1/r3 (791 bp). (D) PCR results for the confirmation of *CDC11* gene disruption. Left: Products amplified by the primer set f3/HPH-chk-f1 (871 bp). Middle: Products amplified by the primer set f4/r4 (567 bp). Right: Products amplified by the primer set HPH-chk-r1/r3 (863 bp). (E) PCR results for the confirmation of *CDC12* gene disruption. Left: Products amplified by the primer set f3/HPH-chk-f1 (812 bp). Middle: Products amplified by the primer set f4/r4 (607 bp). Right: Products amplified by the primer set HPH-chk-r1/r3 (755 bp). M, 100 bp DNA ladder marker; W1, wild type (*MAT1-2*); D1, null mutant (*MAT1-2*); W2, wild type (*MAT1-1*); D2, null mutant (*MAT1-1*); N, negative control (TE buffer).

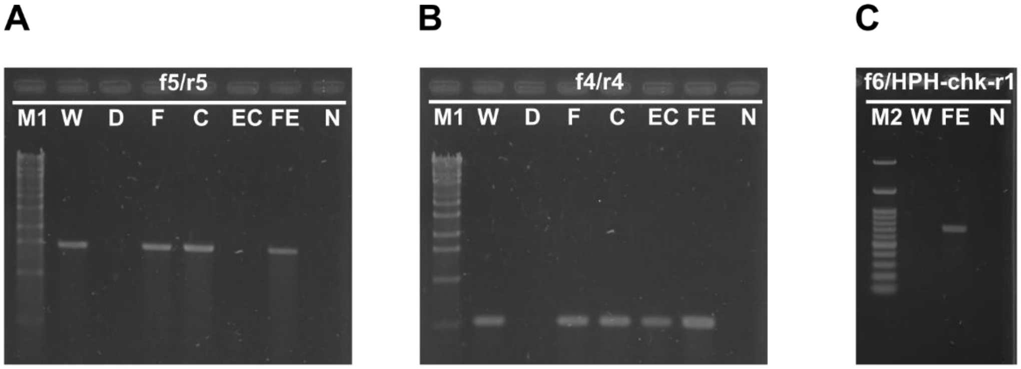

Supplementary Figure S2. PCR results for confirmation of the introduction of several *CDC10* cassettes. (A) Products amplified by the primer set f5/r5 (W, F, C, and FE: 1438 bp). (B) Products amplified by the primer set f4/r4 (W, F, C, EC, and FE: 453 bp). (C) Products amplified by the primer set f6/HPH-chk-r1 (FE: 730 bp). M1, λ/*Sty*I digest; M2, 100 bp DNA ladder marker; W, wild type; D, null mutant; F, *cdc10*^{fs}; C, *CDC10*^{comp}; EC, *CDC10*^{ect}; FE, *CDC10*^{fsect}; N, negative control (TE buffer). The location of primers is displayed in Figure 2.

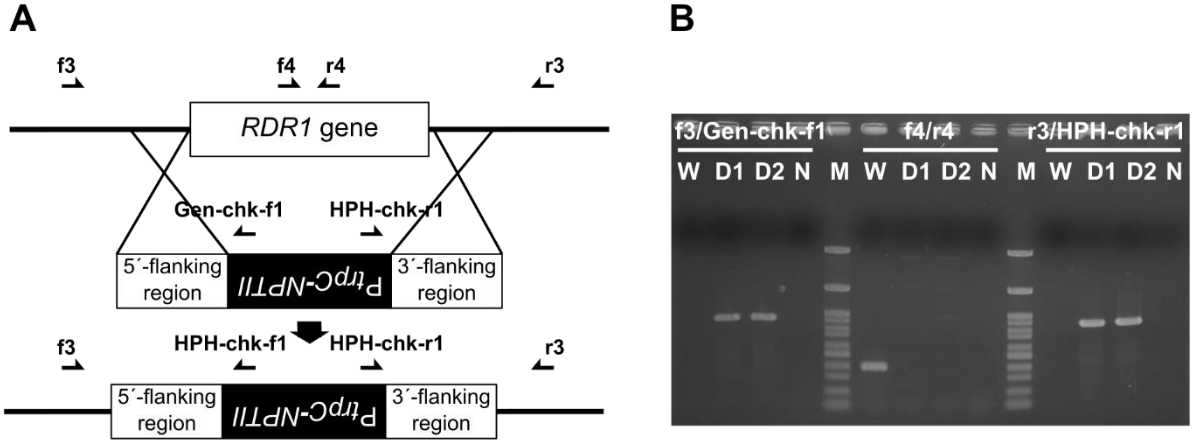

Supplementary Figure S3. Disruption of the *RDR1* genes in *B. maydis*. (A) Schematic illustration of the disruption of three genes and the locations of primers. (B) PCR results for the confirmation of *RDR1* gene disruption. Left: Products amplified by the primer set f3/Gen-chk-f1 (943 bp). Middle: Products amplified by the primer set f4/r4 (404 bp). Right: Products amplified by the primer set HPH-chk-r1/r3 (900 bp). M, 100 bp DNA ladder marker; W, wild type; D1, null mutant 1; D2, null mutant 2; N, negative control (TE buffer).
